# Against All Odds: A Syrian’s Triumph Over Visa Adversity During the COVID-19 Pandemic

**DOI:** 10.1016/j.jaccas.2023.102119

**Published:** 2023-11-14

**Authors:** Anas Hashem, Amani Khalouf

**Affiliations:** Department of Internal Medicine, Rochester General Hospital, Rochester, New York, USA

**Keywords:** cardiology, internal medicine residency, motivation, USA visa

**Figure:**
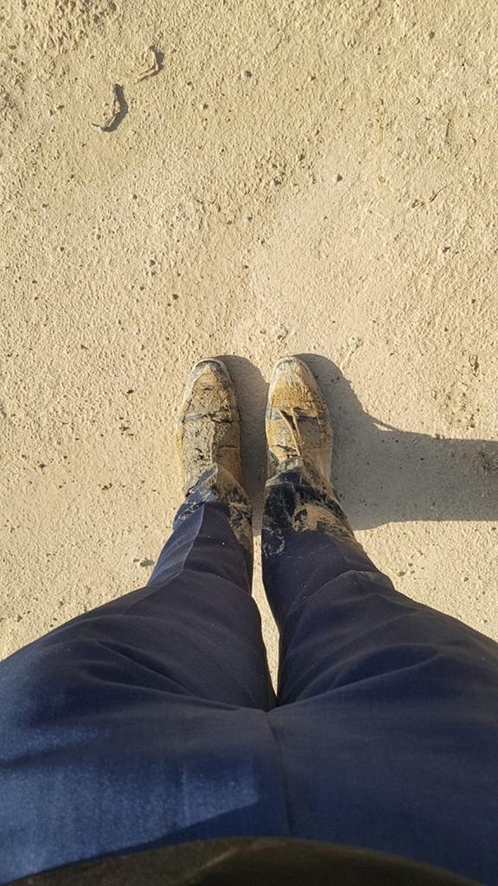

The application process for a United States visa has been and continues to be a significant struggle for foreign physicians.^1^ In August 2019, after completing medical school at the University of Sharjah, I started my medical internship in Sharjah, United Arab Emirates (UAE). Memories of uncertainty and hopelessness still linger. Being Syrian, I was unsure whether my professional path would allow me to ultimately train in the United States or the United Kingdom. In November, I reached out to 100 research-oriented doctors at the Mayo Clinic for an unpaid research trainee position. Only 1 invitation for a “job” interview was extended, followed by an acceptance. With the prospect on the horizon of securing my J-1 visa, an unforeseen global pandemic, COVID-19, suddenly threw my plans into disarray when the US embassy closed access. My dream of training in the United States had been dashed by the weight of the executive order to ban travel for foreign nationals from 7 countries, including Syria.^2^

A couple of months later, Mayo documents arrived via FedEx. I still remember the complex tapestry of emotions, ranging from unbridled joy to lingering uncertainty and even a touch of sadness, knowing that the U.S. embassy was out of reach because of the pandemic. However, in May 2020, embassies tentatively began to reopen, albeit with restrictions. An interview at the U.S. embassy was swiftly granted for the following month just before my Mayo Clinic starting date. Finally, there was a glimmer of light at the end of the tunnel! Yet, Abu Dhabi's unyielding quarantine measures were a major obstacle because the city, with its 3 entrances heavily fortified by a military presence, was impassable, regardless of COVID-19 status.

With steadfast faith, I gathered my documents, including my Syrian passport, and began a journey that would challenge my endurance. It was a journey imbued with an unmistakable air of surrealism: the audacity of crossing the desert to meet my destiny. The day before my appointment, I adorned myself in my finest attire, a testament to my unwavering commitment to professionalism, whether facing a checkpoint officer or a U.S. embassy interviewer. I undertook a 2.5-hour drive to the first of 3 checkpoints. The military presence was more than I had anticipated. I outlined my predicament to the officer, and his response still echoes: “If you were the U.S. embassy consul, you wouldn't be allowed in. GO BACK TO DUBAI!” The shock was profound. I hadn't foreseen this particular barrier, having surmised that securing a visa waiver would be the greatest challenge. Nevertheless, my next move seemed almost preordained. I proceeded to the subsequent exit and was again promptly redirected back to Dubai.

I was determined to try one last gambit: the “Last Exit” from Dubai to Abu Dhabi. The line was interminable, necessitating a 2-hour wait before I reached the officers. When my turn came, I presented my documents and implored the officers to consider my case earnestly. The junior officer conferred with his senior, and after a brief wait, they relayed what I had dreaded: “Go back to Dubai.”

I drove away from the checkpoint, my heart heavy with disappointment. Just beyond the checkpoint and before the exit bridge to Dubai, I saw a small restaurant with a parking lot. It provided me with a refuge where I could gather my thoughts. I realized in that moment that my Syrian origin was an indelible part of my identity, irrespective of my physical location. This epiphany showed me that my struggles were just a fraction of what Syrian refugees endure.

I committed to taking the arduous path and traversed the unforgiving terrain of Abu Dhabi’s desert during a searing June heat wave, with temperatures soaring between 45 °C and 50 °C. I trekked nearly 5 kilometers before finally stumbling upon a taxi permitted through the checkpoint. Miraculously, I went unnoticed, or perhaps I was noticed and allowed to pass. As I finally set foot in Abu Dhabi, relief splashed over me, mingling with the sheer elation of being a step closer to realizing my dreams while covered with mud (Figure 1). The following morning, at the U.S. embassy, I asked myself whether I would be allowed entry or sent back to the desert. When my interview started, I maintained a mask of calm, hiding the palpitations that threatened to betray my composure. I met each question with a measured response, supported by the evidence I held in my trembling hands. When the interview concluded, a rejection letter lay before me, a shocking denouement.

**Figure 1:**
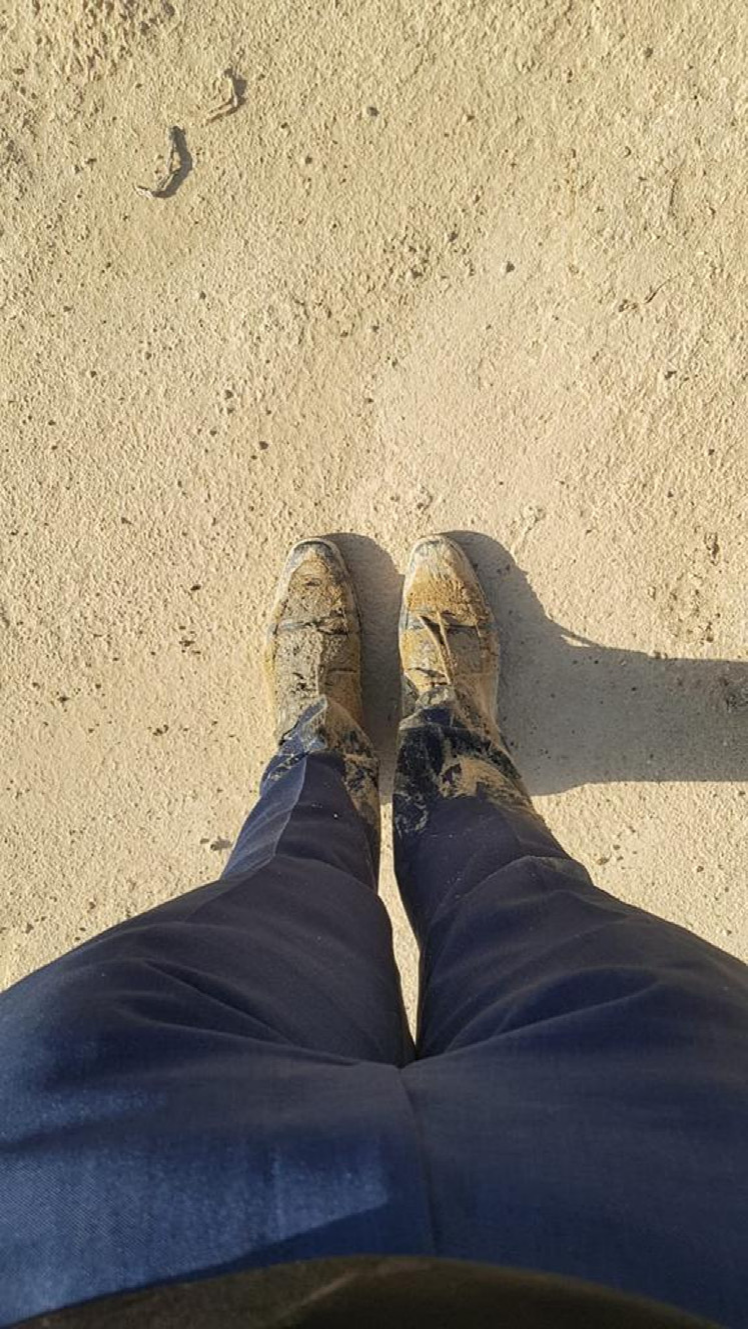
Covered With Mud After I Had Walked 5 Kilometers in the Desert

At that moment, I humbly asked the interviewer for an explanation. I had provided every shred of evidence, and my future hung in the balance. “Why?” I implored. “I have all the evidence—and what about my job promise in the United States?” He replied: “Sorry, but you are not qualified based on Presidential Proclamation 9645. Read the letter I share with you.” (Figure 2). Leaving the embassy, I was awash with extreme disappointment and frustration. Now what? Was this golden opportunity slipping through my fingers? Was it all worth this agony? Were there other options? I said to myself, “Anas, if you were a true refugee, you would be far from your parents, destitute, and bereft of a future.” Perhaps refugees harbor a reservoir of resilience and faith that I had yet to tap into!

**Figure 2:**
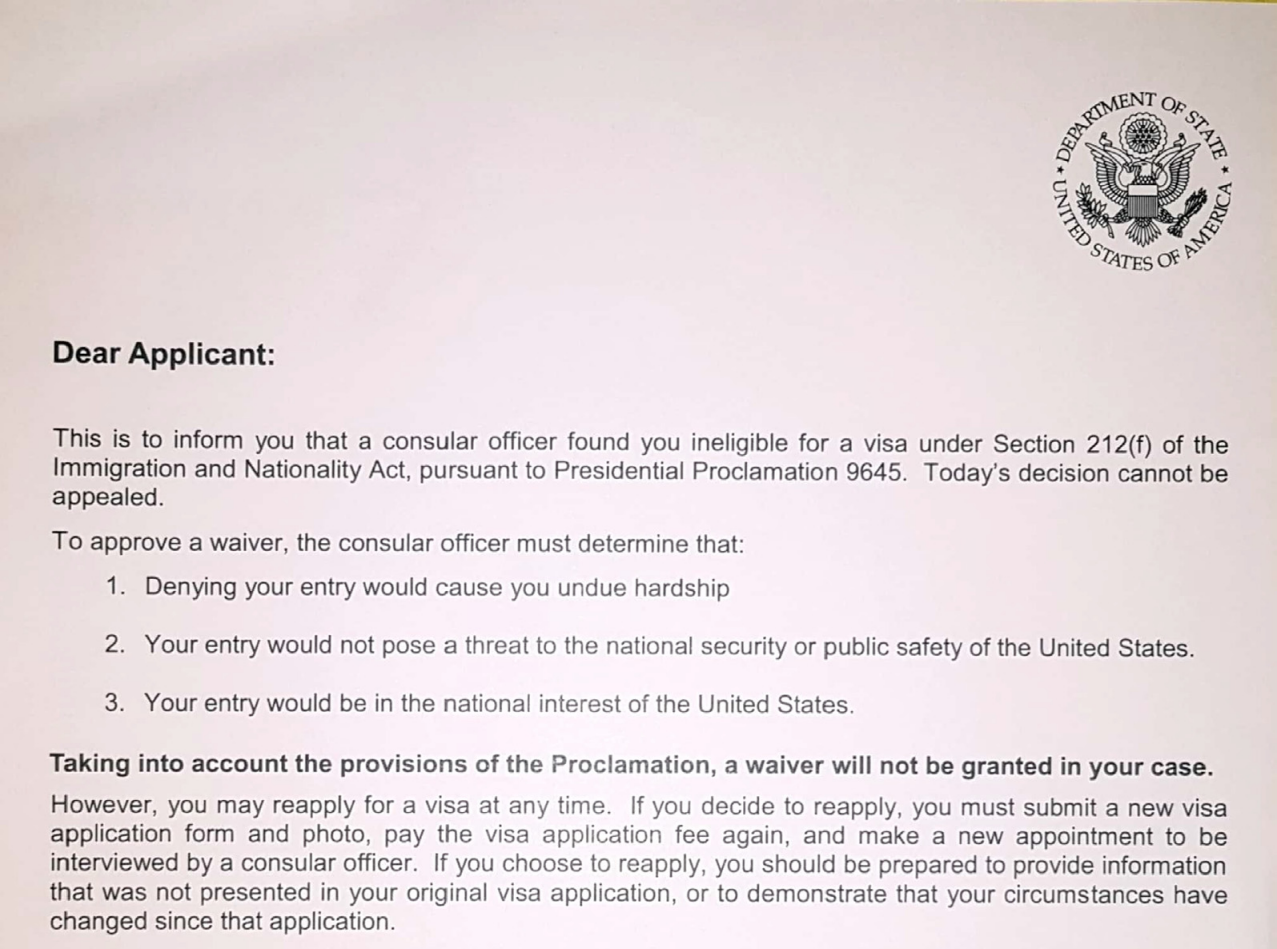
Rejection Letter Based on Presidential Proclamation 9645

I stood before the U.S. embassy, 100 kilometers from my car, with only $13 after spending all my savings on a visa appointment. I had no way to reach my car with the money I had. Suddenly, a poor Yemeni man, who had been unemployed because of the pandemic, offered to help. He drove me to a gas station 10 kilometers from the checkpoint, keeping only $10 and leaving me with $3 for water. The heat was unbearable; it felt more like 80 °C. I soon was drenched in sweat, my mind muddled, my breaths shallow, and my heart racing. Was it the result of dehydration, the fear of being intercepted by officers as I crossed the desert, or the dread of perishing alone, with no one aware of my whereabouts? After nearly 2 hours, I finally reached my car. You can scarcely imagine the surge of emotion that coursed through me.

Upon returning to my university dormitory, I opened my e-mail and composed a lengthy missive to the U.S. embassy, ending with this plea: “All I am asking for is a fair evaluation of my waiver eligibility based on U.S. rules that consider my personal circumstances, rather than a national exclusion. I am a human being before being a Syrian and my application should be taken into consideration like any other applicant.” At that moment, I decided to consult a lawyer, and I was able to connect with Muna Jundy, who raised my hopes by crafting my waiver request. Once again, I applied for a visa appointment, given that the Abu Dabai checkpoints were now open to those with a negative COVID-19 test result (no more desert crossing!) My interview went well, and I answered each query to the best of my ability, with evidence. With a nod, the interviewer informed me that I would be subject to administrative processing—a term that, in visa parlance, is known as a “black hole” and carries a shroud of uncertainty. Sometimes, living with uncertainty, tinged with a glimmer of hope, is preferable to a definitive, unyielding dismissal.

Days later, I received notification that my U.S. visa was approved, sparking immense joy, even though my future position was unpaid. Yet, this was America, the land of boundless opportunity! Meanwhile, what if a paid residency position in the UAE was offered? My parents hoped for financial support. The UAE match results coincided with my visa approval. An e-mail revealed I had not matched the UAE residency program despite achieving the 100th percentile on the Emirates medical residency entrance examination. A strange mix of elation and sadness washed over me because my efforts and achievements seemed overlooked in my homeland. Here, origins, nationality, and your passport hold the most sway.

Mercifully, my future had been decided, a path laid out before me—my destiny was in the United States! Even though my job was unpaid, I was privileged to receive a $3500 scholarship from the Syrian American Medical Society, which provided me with an acceptable fund to cover my stay, as well as some financial support from my family and relatives to buy groceries and food. My journey began with clinical research training at the Mayo Clinic, guided by Dr Andres Acosta, to whom I owe a profound debt of gratitude. I was responsible for creating and assessing clinical trial protocols, enrolling and gaining consent from research participants, managing and analyzing data, and composing research manuscripts. During this period, I actively participated in numerous clinical and retrospective studies, some of which have already been featured in prestigious journals with high impact factors. I took on the role of primary author for 2 of these studies and contributed as a co-author on others. Two years ago, I started my internal medicine residency training at Rochester General Hospital, aspiring to further advance my career in cardiology. I met the requirements for this role by earning my MD degree, achieving commendable scores in both Step 1 and Step 2 of the United States Medical Licensing Examination, acquiring clinical experience in the United States, and boosting my curriculum vitae through clinical research training and publication.

In sharing my story, I emphasize that there are many challenges in life. You never know when the tide might turn in your favor. As the Arabic saying goes, “Those who keep knocking on doors, they will, one day, find them open.”

## Funding Support and Author Disclosures

The authors have reported that they have no relationships relevant to the contents of this paper to disclose.
